# The Myb-related protein MYPOP is a novel intrinsic host restriction factor of oncogenic human papillomaviruses

**DOI:** 10.1038/s41388-018-0398-6

**Published:** 2018-07-17

**Authors:** Elena Wüstenhagen, Fatima Boukhallouk, Inka Negwer, Krishnaraj Rajalingam, Frank Stubenrauch, Luise Florin

**Affiliations:** 1 Department of Medical Microbiology and Hygiene, University Medical Center of the Johannes Gutenberg University Mainz, Obere Zahlbacher Strasse 67, Mainz, 55131 Germany; 2 Max Planck Institute for Polymer Research, Ackermannweg 10, Mainz, 55128 Germany; 3 Molecular Signaling Unit, University Medical Center of the Johannes Gutenberg University, Mainz, Germany; 4 Division of Experimental Virology, Institute for Medical Virology and Epidemiology of Viral Diseases, University Hospital, Tübingen, Germany

## Abstract

The skin represents a physical and chemical barrier against invading pathogens, which is additionally supported by restriction factors that provide intrinsic cellular immunity. These factors detect viruses to block their replication cycle. Here, we uncover the Myb-related transcription factor, partner of profilin (MYPOP) as a novel antiviral protein. It is highly expressed in the epithelium and binds to the minor capsid protein L2 and the DNA of human papillomaviruses (HPV), which are the primary causative agents of cervical cancer and other tumors. The early promoter activity and early gene expression of the oncogenic HPV types 16 and 18 is potently silenced by MYPOP. Cellular MYPOP-depletion relieves the restriction of HPV16 infection, demonstrating that MYPOP acts as a restriction factor. Interestingly, we found that MYPOP protein levels are significantly reduced in diverse HPV-transformed cell lines and in HPV-induced cervical cancer. Decades ago it became clear that the early oncoproteins E6 and E7 cooperate to immortalize keratinocytes by promoting degradation of tumor suppressor proteins. Our findings suggest that E7 stimulates MYPOP degradation. Moreover, overexpression of MYPOP blocks colony formation of HPV and non-virally transformed keratinocytes, suggesting that MYPOP exhibits tumor suppressor properties.

## Introduction

The physical and chemical barrier of the skin mediates the first line of defense against invading pathogens. This is additionally supported by the cellular innate immunity [1]. Here, so-called restriction factors detect invading viruses and restrict the viral replication cycle at different stages [1, 2]. For HPV, it is known that a microwound is required to overcome the physical barrier function of the skin to gain access to mitotically active basal cells of the epithelium [3, 4]. However, only a limited number of cellular factors that restrict the viral replication cycle in these cells are identified so far [1, 5].

Human papillomaviruses are small DNA viruses associated with a wide range of benign and malignant epithelial tumors. These viruses account for 5.2% of the worldwide cancer burden [6]. Persistent infections with certain HPV types are the main causative agents for cervical cancer (e.g. HPV16 and 18) and genital warts (e.g. HPV6 and 11) [7, 8]. The virus consists of two capsid proteins comprising the major capsid protein L1 and the minor capsid protein L2, and a double-stranded DNA genome. The genome is subdivided into an early region, a late region, and a noncoding region, the so-called long control region (LCR) or upstream regulatory region [3, 4]. The LCR encompasses about 850 bp containing the major transcription start site of the early promoter, enhancer, and silencer elements. Regulation of early gene expression including expression of the oncogenes E6 and E7 is mediated by binding of cellular and viral factors to the LCR [9–15].

The viral replication cycle starts with virus entry via endocytosis [16–18]. After internalization, the capsid is disassembled in endosomes [19] and the multifunctional L2 protein directs the viral DNA (vDNA) into the host cell nucleus [20–24]. Once infection is established in basal keratinocytes, viral transcription and replication is regulated by the counterbalance of activating and repressing host and viral proteins [5, 7, 10, 25, 26]. The low basal activity of the HPV LCR and control of the early HPV genes facilitates persistent infection [5, 27]. Expression of the early viral oncogenes results in increased cell division by inactivating key cellular players, which are involved in the regulation of apoptosis and cell cycle control [28]. Uncontrolled oncogene expression eventually leads to tumor progression [29–32]. Studies in recent years have uncovered a number of proteins that contribute to intrinsic cellular immunity and tumor suppression by HPV early gene restriction [3, 5, 7]. However, further investigations are needed to identify novel antiviral proteins and to better understand the counterbalance between viral proteins and cellular restriction factors and/or tumor suppressors.

In this study, we identified the so far unknown human Myb-related transcription factor (TF), MYPOP, as a novel interaction partner of the minor capsid protein L2 and the viral DNA. It was shown that MYPOP’s orthologous murine protein p42^POP^ is able to repress the consensus Myb recognition element (MRE) when introduced into the minimal herpesvirus thymidine kinase promoter [33]. We uncovered that MYPOP acts as a restriction factor for the oncogenic HPV types 16 and 18 as it represses the LCR activity of both viruses. Furthermore, MYPOP mediates reduction of HPV16 E6*I and E1^E4 early gene transcripts. Interestingly, total MYPOP amounts are strongly reduced in diverse HPV-transformed cell lines and cervical cancer, suggesting MYPOP downregulation as a precondition for oncogene expression, which is required for proliferation and finally tumor progression. Accordingly, overexpression of MYPOP resulted in a substantial decrease in the number of HPV16- or HPV18-immortalized and non-virally transformed keratinocytes, indicating that MYPOP is able to block proliferation of tumor cells.

## Results and discussion

### MYPOP is a novel interaction partner of the HPV16 L2 protein and the viral DNA

**Fig. 1:**
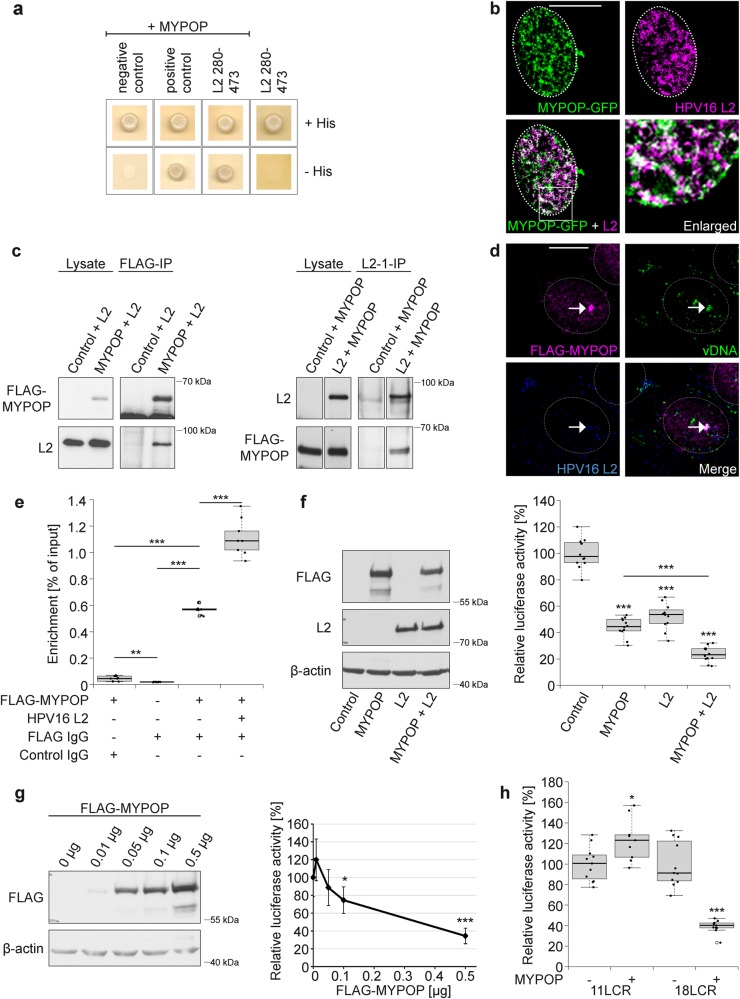
MYPOP forms a complex with viral components and represses the LCR of oncogenic HPV types. **a–e** MYPOP is a novel interaction partner of the minor capsid protein L2 and the viral DNA. **a** Yeast strain L40 expressing a negative control (LexA-lamin), a positive control (LexA-Fos2), or the bait LexA-L2 280-473 fusion construct was transformed with corresponding prey construct B42-HA-MYPOP. Transformants were tested for the prototrophic marker histidine (−His) reporting bait–prey interaction. **b** HaCaT cells were co-transfected with MYPOP-GFP (green) and L2 (magenta). **c** HaCaTs were co-transfected with pcDNA3.1 or HPV16 L2 and FLAG-tagged MYPOP or empty FLAG vector as indicated. Western blot of lysates show protein expression. Immune complexes were precipitated and detected with a specific FLAG or L2 antibody. MYPOP shows a migration behavior of about 60 kDa in the SDS-PAGE. **d** HaCaTs were transfected with FLAG-MYPOP and then incubated with EdU-labeled HPV16 LCR PsV for additional 24 h. Cells were processed for detection of EdU-labeled vDNA (green), FLAG-MYPOP using polyclonal rabbit FLAG (magenta) and L2 (blue). **e** Chromatin from HPV18-transformed HeLa cells was prepared after transfection with FLAG-MYPOP, FLAG-MYPOP and HPV16 L2, or control FLAG vector as indicated. Binding of MYPOP to the integrated LCR of HPV18 was analyzed by ChIP using a FLAG-specific or control mouse IgG antibody. Control ChIP with anti-FLAG antibody was also performed with the lysate of control-transfected cells. Precipitated and purified DNA served as a template for qPCR using HPV18 LCR primers. Part of the total chromatin was used as input. Changes were expressed as the ratio of immunoprecipitated chromatin target from MYPOP-transfected to control-transfected cells enriched with respect to input chromatin. The experiment was repeated three times and shown is one representative experiment. Data (*n* = 9) were analyzed using Welch two-tailed *t*-test: *p* = 0.00297, *t* = −4.1924, dF = 8.0695 (Control IgG–Anti-FLAG w/o MYPOP), *p* = 5.187 × 10^−11^, *t* = −46.091, dF = 8.0191 (Anti-FLAG w/o MYPOP–Anti-FLAG w/MYPOP) or two-tailed unpaired *t*-test *p* = 2.2 × 10^−16^, *t* = −38.898, dF = 16 (Control IgG–Anti-FLAG w/MYPOP) or Welch two-tailed *t*-test *p* = 6.222E^−7^, *t* = −12.056, dF = 9.1745 (Anti-FLAG w/MYPOP–Anti-FLAG w/MYPOP + L2). **f–j** L2 represses the LCR activity of oncogenic HPV types. **f** HaCaTs were transfected with 0.5 µg pGL4.20 luciferase reporter vector containing the long control region of HPV16. The cells were co-transfected with either control vector, FLAG-MYPOP, L2, or FLAG-MYPOP together with L2 as indicated and lysed to monitor protein expression by western blot (left panel). Luciferase activity was measured and normalized to lactate dehydrogenase (LDH) activity (right panel). Control-transfected cells were set to 100% and data (*n* = 12) were analyzed using two-tailed unpaired *t*-test: *p* = 6.74 × 10^−13^, *t* = 14.766, dF = 22 (Control–MYPOP), *p* = 1.103 × 10^−10^, *t* = 11.375, dF = 22 (Control–L2) or Welch two-tailed *t*-test *p* = 1.866 × 10^−13^, *t* = 21.256, dF = 16.566 (Control–MYPOP + L2) or two-tailed unpaired *t*-test *p* = 1.023 × 10^−7^, *t* = 7.7372, dF = 22 (MYPOP–MYPOP + L2). **g** HaCaTs were transfected with 0.5 µg pGL4.20 luciferase reporter vector containing the long control region of HPV16 and co-transfected with increasing amounts of FLAG-MYPOP as indicated. MYPOP expression is shown by western blot (left panel). Luciferase activity was measured as in **f** (right panel). The values obtained from two independent experiments are given as mean ± SD and the mean for pGL4.20 HPV16 LCR with empty FLAG vector was set to 100%. Data (*n* = 7) were analyzed using two-tailed unpaired *t*-test: *p* = 0.1257, *t* = 1.6461, dF = 12 (0.01 µg), *p* = 0.3287, *t* = 1.0182, dF = 12 (0.05 µg), *p* = 0.02428, *t* = −2.576, dF = 12 (0.1 µg) or Welch two-tailed *t*-test: *p* = 7.447 × 10^−5^, *t* = 7.4882, dF = 7.9069 (0.5 µg). **h** HaCaTs were co-transfected with pGL4.20 HPV11 LCR (11 LCR) or HPV18 LCR (18 LCR) together with control FLAG vector or FLAG-MYPOP. Influence of MYPOP on LCR was measured as above. The pGL4.20 11 LCR with empty FLAG vector or pGL4.20 18 LCR with empty FLAG vector was set to 100%. Data were analyzed using two-tailed unpaired *t*-test: *p* = 0.01191, *t* = −2.7971, dF = 18 (11 LCR, *n* = 9–11) or Welch two-tailed *t*-test *p* = 2.005 × 10^−6^, *t* = 8.7196, dF = 11.565 (18 LCR, *n* = 11). The values obtained from three independent experiments are given as boxplots (**e**, **f**, **h**). Monoclonal mouse antibody L2-1 and monoclonal mouse FLAG antibody was used (**b**, **c**, **f**, **g**). Due to clarity and conciseness the western blot images are cropped (**c**, **f**, **g**). Scale bar = 10 µm and nuclei are shown with dotted lines (**b**, **d**). The lower panel shows β-actin as a loading control (**f**, **g**). *p ≤ 0.05, **p ≤ 0.01; ***p ≤ 0.001 (**e**–**h**)

During HPV entry, the minor capsid protein L2 accompanies the viral DNA into the host cell nucleus and is accessible to cytoplasmic and nuclear proteins [20, 23, 24]. Using a Y2H screening approach, we identified novel interaction partners of the HPV16 L2 protein [14, 34–36]. Among others, the Myb-related TF MYPOP was discovered (Fig. 1a) [35]. As this is the first study of human MYPOP, we characterized endogenous, overexpressed and purified MYPOP in immunofluorescence and western blot (WB) analyses (Suppl. Figure 1). MYPOP showed nuclear and cytoplasmic localization and WB bands of about 60 kDa. Co-localization and interaction analyses verified interaction of L2 and MYPOP (Fig. 1b–d). Moreover, we detected association of the incoming virally transduced DNA with the TF MYPOP in the nucleus of infected cells (Fig. 1d).

Chromatin immunoprecipitation (ChIP) studies in HeLa cells, which are transformed by oncogenic HPV and harbor the integrated viral genome within the host cell genome [37] revealed binding of MYPOP to the LCR of the HPV genome (Fig. 1e). The interaction of the TF to the HPV LCR was additionally strengthened in the presence of L2. It has been shown that only L2 is accessible to cytoplasmic or nuclear proteins during virus entry and that the viral genome remains protected in a vesicular structure until the loss of the limiting membrane in the newly formed nucleus [38, 39]. Our finding suggests that MYPOP senses incoming HPV16 by binding to the capsid protein L2 and forms a tripartite complex with the viral LCR to modulate viral gene expression.

### MYPOP silences the LCR of oncogenic HPV types

Next, we investigated whether MYPOP directly regulates the activity of the HPV16 LCR by performing promoter-reporter gene assays. Non-virally transformed HaCaT keratinocytes were co-transfected with the promoter-reporter plasmid pGL4.20 containing the HPV16 long control region (pGL4.20 HPV16 LCR), MYPOP and/or L2 expression vectors (Fig. 1f). Measurements of the LCR activity (analyzed by relative luciferase activity) clearly demonstrated that MYPOP acts as transcriptional repressor of the HPV16 LCR. Again, L2 significantly enhanced the repressive effect (Fig. 1f). Transfection of increasing amounts of MYPOP revealed that MYPOP functions in a concentration-dependent manner (Fig. 1g). Likewise. earlier studies on p42^POP^ had showed that the MYPOP’s orthologous murine protein represses transcription [33], suggesting a highly conserved mechanism of gene regulation.

Direct binding of Myb TFs to viral and cellular promoters is a well-characterized interaction of the DNA-binding domain (DBD) with the MRE [40–42]. Our analyses using MYPOP mutants demonstrated that the predicted DBD of MYPOP is able and sufficient to repress the HPV16 LCR while; the C-terminal part of MYPOP was inactive (Suppl. Figure 2a–c). The MRE comprises a 5′-AAC-3′ core sequence, flanked by a pyrimidine at the 5′-end, and a guanine or thymine at the 3′-end [40, 41, 43]. We identified seven putative binding sites (PyAACG/T) in the LCR of HPV16 (Suppl. Figure 2d). Using different LCR constructs, we found that one binding motif located 85 bp upstream of the transcription start site is sufficient to silence the LCR activity, whereas removal of all putative binding sites led to a loss of MYPOP-mediated LCR repression (Suppl. Figure 2e).

Next, we uncovered that the MYPOP-mediated silencing of the LCR is not limited to HPV16, but was also observed for the high-risk HPV18 LCR, which comprises three MREs (Fig. 1h and Suppl. Figure 2f). By contrast, the low-risk type HPV11 was not repressed although it exhibits one putative MRE. This sequence may not function as a MYPOP binding site or the long distance of the MRE to the p1 promoter/TATA box may explain the MYPOP resistance of HPV11. In line with this observation, it has been shown that regions located more at the 5′-end of the HPV11 LCR play no or minor roles on transcriptional activity [44]. One might speculate that the observed sensitivity of high-risk HPV to MYPOP and, therefore, reduced early gene expression supports prolonged persistence. Time to clearance is 12–18 months for high-risk HPV and 4–9 months for low-risk HPV [8].

### MYPOP restricts HPV16 PsV infection

**Fig. 2:**
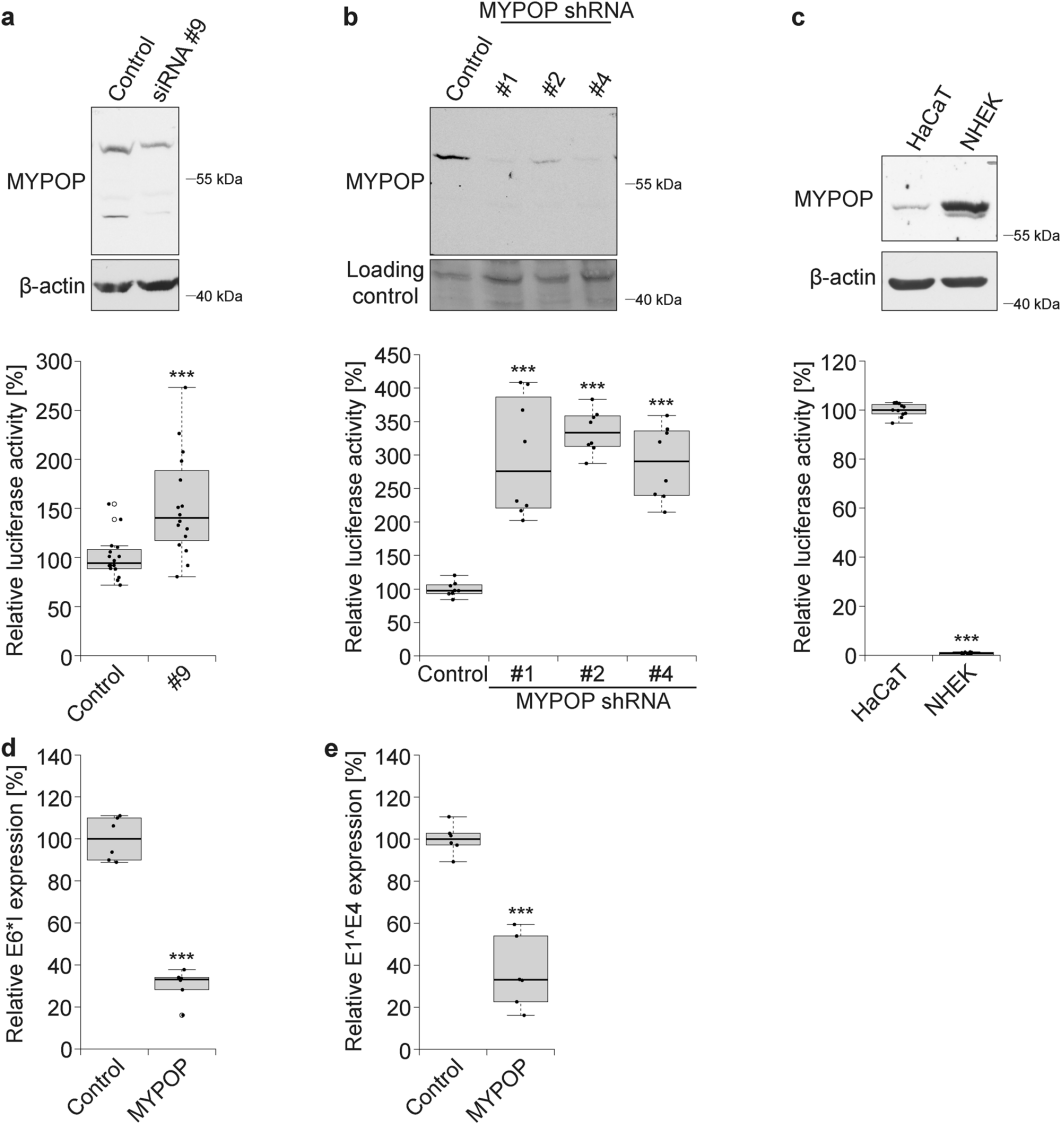
MYPOP protein level negatively correlates with HPV16 PsV infection and early gene expression. **a–c** MYPOP in HPV16 infection assay. **a** HaCaTs were transfected with control siRNA or MYPOP-specific siRNA #9 for 48 h and then re-transfected for additional 48 h. Seventy-two hours after initial siRNA transfection, knockdown efficiency of MYPOP was analyzed by western blot (upper panel) or infected with HPV16 LCR PsV (lower panel). Relative luciferase activity as measure for infection was assessed 24 h later and normalized to lactate dehydrogenase (LDH) measurements. Control siRNA-treated cells were set to 100% and data (*n* = 16) were analyzed using Wilcoxon rank sum test: *p* = 0.0004666, *W* = 39 (#9). **b** HaCaT cells were transduced with lentiviruses containing MYPOP-specific shRNAs. Knockdown efficiency (upper panel) and relative luciferase activity (lower panel) were measured as in **a**. Control shRNA-treated cells were set to 100% and data (*n* = 8) were analyzed using Welch two-tailed *t*-test: *p* = 0.0003732, *t* = −6.2553, dF = 7.216 (#1), *p* = 1.792 × 10^−8^, *t* = −19.682, dF = 8.6327 (#2), *p* = 1.893 × 10^−5^, *t* = −9.4527, dF = 7.5528 (#4). **c** Western blot analysis of MYPOP expression levels in HaCaT and NHEK cells (upper panel). HaCaT and NHEK cells were infected with HPV16 LCR PsV. Luciferase activity as a measure of infectivity was assessed 24 h later and normalized by LDH measurement. HaCaT cells were set to 100% and data (*n* = 11) were analyzed using Wilcoxon rank sum test *p* = 2.835 × 10^−6^, *W* = 121. **d, e** MYPOP in HPV16 early gene expression. **d** SCC-13 cells were co-transfected with re-circularized HPV16 wt (114/B) and FLAG-MYPOP or empty FLAG vector. After 48 h of transfection, total cellular RNA was isolated and analyzed for HPV16 E6*I spliced early transcripts. Control-transfected cells were set to 100% and data (*n* = 6) were analyzed using two-tailed unpaired *t*-test: *p* = 1.067 × 10^−7^, *t* = 13.348, dF = 10 (E6*I). **e** Experiments were performed as described for panel **d**, but analyzed for HPV16 E1^E4 spliced early transcripts. Control-transfected cells were set to 100% and data (*n* = 6) were analyzed using two-tailed unpaired *t*-test: *p* = 7.417 × 10^−6^, *t* = 8.4307, dF = 10 (E1^E4). The values obtained from three (or two for **b**) independent experiments are given as boxplots (**a–c** lower panel, **d**, **e**). Detection of endogenous MYPOP was performed using polyclonal MYPOP antibody (Abcam) (**a**–**c**). Due to clarity and conciseness the western blot images are cropped (**a–c** upper panel). The lower panel shows β-actin as a loading control (**a**, **c**). ***p ≤ 0.001 (**a–e**)

The repressive effect of MYPOP on the transfected HPV LCR was extended to incoming histone associated HPV-transduced DNA. For this study, we replaced the viral genome by the pGL4.20 HPV16 LCR plasmid, thereby generating HPV16 LCR PsV. Next, we reduced the endogenous MYPOP level in HaCaT cells by RNA interference. These experiments provided deeper insight into the nature of the TF (Fig. 2 and Suppl. Figure 3). First, four different MYPOP-specific siRNAs led to a reduction of MYPOP mRNA levels without affecting protein amounts and infection when tested 48 h after siRNA transfection (Suppl. Figure 3). Extended incubation times to 4 or 7 days with MYPOP-specific siRNA combined with re-transfection of the siRNA after 48 h caused a reduction of the endogenous MYPOP protein (Fig. 2a). These findings indicate an unexpected high stability and low turnover rate of the TF and might explain why MYPOP was not detected earlier by siRNA screening. To further increase knockdown efficiency, we used a lentiviral RNAi system. Incubation times of more than 1 week were considerably more successful: all shRNA constructs led to a decrease of MYPOP protein and infectivity was increased by 300–400% compared to control shRNA-treated cells (Fig. 2b), corroborating the antiviral activity of MYPOP on incoming viral DNA.

To verify the negative correlation of the MYPOP expression level and HPV16 PsV infection in different cells, we tested total cellular MYPOP amounts and infection efficiencies of HaCaT cells and primary keratinocytes (NHEK) (Fig. 2c). These results uncovered higher MYPOP expression levels and lower infection rates of NHEK when compared to HaCaT cells. Our data again provide strong evidence that MYPOP limits the infection of human skin cells by HPV16 and acts as a viral restriction factor. Interactions of this factor with other viral promoters may be important in mediating intrinsic immunity against additional viruses.

### MYPOP potently silences HPV16 early gene expression

**Fig. 3:**
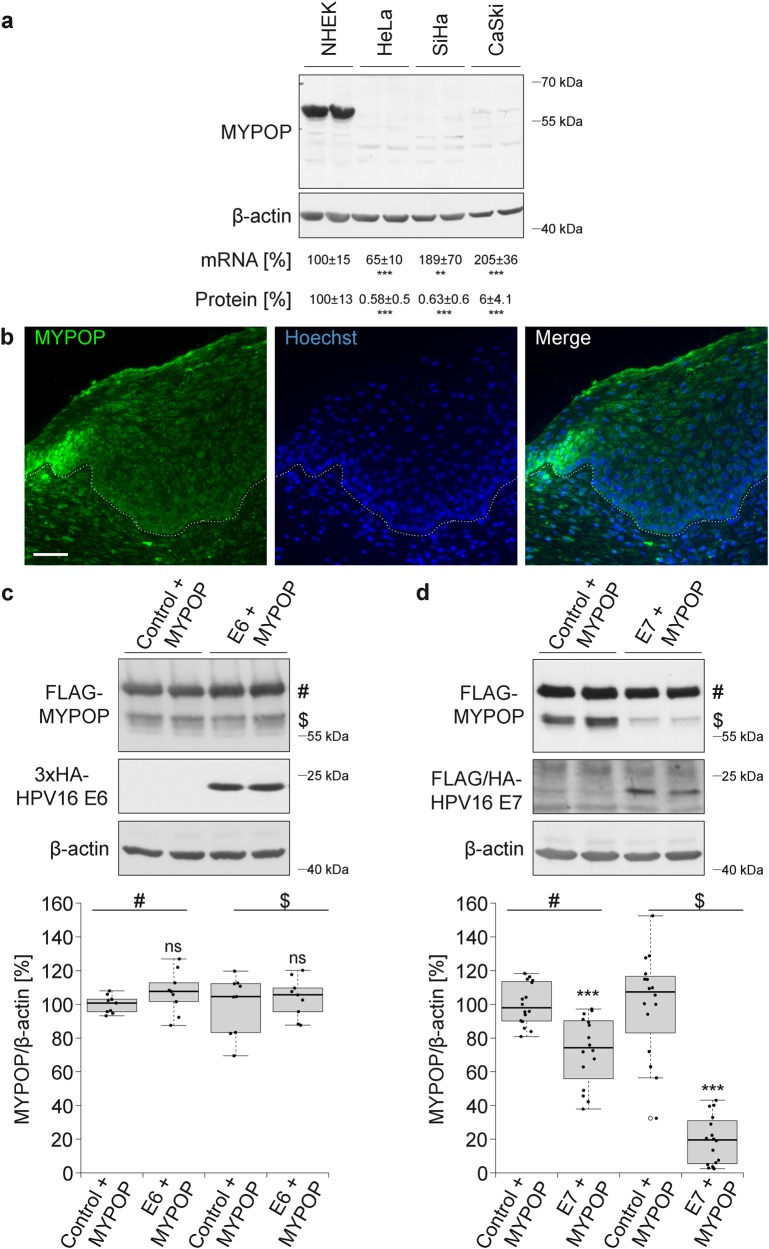
MYPOP is reduced in HPV-transformed cell lines and cancer tissue. **a** Quantification of MYPOP protein and mRNA in primary keratinocytes (NHEK) and HPV-transformed cells lines HeLa (HPV18), SiHa, and CaSki (both HPV16). Total cellular mRNA was analyzed by quantitative real-time PCR (qPCR). NHEK were set to 100% and data (*n* = 6) were analyzed using two-tailed unpaired *t*-test: *p* = 0.000944, *t* = −4.6245, dF = 10 (HeLa) or Welch two-tailed *t*-test *p* = 0.01839, *t* = −3.3236, dF = 5.4495 (SiHa) or two-tailed unpaired *t*-test *p* = 6.653 × 10^−5^, *t* = 6.5284, dF = 10 (CaSki). Densitometric quantification of the western blots (a representative western blot is shown in the upper panel) was performed with ImageJ software and relative MYPOP band intensities were normalized to β-actin. NHEK cells were set to 100% and data (*n* = 5) were analyzed using Welch two-tailed *t*-test *p* = 9.81 × 10^−7^, *t* = −19.968, dF = 6.0255 (HeLa), *p* = 9.71 × 10^−7^, *t* = 19.949, dF = 6.035 (SiHa), *p* = 92.11 × 10^−7^, *t* = −17.647, dF = 7.5346 (CaSki). **b** Expression of MYPOP in human cervical carcinoma in situ. Human cervical tissue sections were stained for MYPOP (green). Nuclei were counterstained with Hoechst (blue). Scale bar = 10 µm. **c** HaCaTs were transfected with FLAG-MYPOP without and with 3xHA16E6 as indicated. The cells were lysed to monitor protein expression by western blot using FLAG and HA antibody. Densitometric quantification of western blot data from **c**. Relative band intensities were assessed by normalizing FLAG-MYPOP levels (# and $) to the β-actin using ImageJ software. Control + FLAG-MYPOP-transfected cells were set to 100% for # and for $. Data (*n* = 9) were analyzed using Welch two-tailed *t*-test: *p* = 0.1431, *t* = −1.5807, dF = 10.68 (#) or two-tailed unpaired *t*-test *p* = 0.5826, *t* = −0.56091, dF = 16 ($). **d** Same as **c**, but cells were transfected with FLAG-MYPOP without and with FLAG-HA-HPV16 E7 as indicated. Densitometric quantification of western blot data was performed as described in **c**, but FLAG-HA-HPV16 E7. Control + FLAG-MYPOP-transfected cells were set to 100% for # and for $. Data (*n* = 16) were analyzed using two-tailed unpaired *t*-test: *p* = 6.714 × 10^−5^, *t* = 4.6244, dF = 30 (#), or Welch two-tailed *t*-test *p* = 3.645 × 10^−9^, *t* = 9.583, dF = 21.241 ($). The values obtained from three (or six for **d**) independent experiments are given as boxplots (**a**, **c**, **d**). Due to clarity and conciseness the western blot images are cropped (**a**, **c**, **d**) and the lower panel shows β-actin as a loading control (**a**, **c**, **d**). # represents FLAG-MYPOP of higher molecular weight; $ represents FLAG-MYPOP of lower molecular weight (**c**, **d**); ns, not significant; **p ≤ 0.01; ***p ≤ 0.001

As our MYPOP-based ChIP and promoter studies demonstrated binding to the HPV LCR and repression of the LCR activity, we controlled the effect of MYPOP in the whole HPV genome context on the expression of viral early genes. We co-transfected squamous cell carcinoma cells SCC-13 [45] with HPV16 wt (isolate 114B) and FLAG-MYPOP or empty FLAG vector. After 48 h, total cellular RNA was isolated and analyzed for HPV16 E6*I and E1^E4 spliced early transcripts (Fig. 3a). Measurement of these transcripts is the standard method to analyze early gene expression of HPV16 [46–48]. Corroborating our promoter-reporter gene assays, MYPOP reduced HPV16 early genes transcription. These findings strongly support the biological relevance of MYPOP as viral restriction factor. Moreover, the transcriptional suppressor efficiently silences LCR activity even in the absence of L2 and this finding suggests that MYPOP may play a role in different steps of the viral replication cycle, including HPV-induced oncogenesis as the HPV LCR likewise controls the viral oncogenes E6 and E7.

### MYPOP is eliminated in HPV-transformed cells

It has been described that early viral oncoproteins E6 and E7, which are expressed in HPV-transformed cells, eliminate detectable tumor suppressors p53 and pRb, respectively [30]. Interestingly, we uncovered that MYPOP protein- but not mRNA levels are almost absent in whole cell lysates of HPV18-transformed HeLa and HPV16-transformed SiHa and CaSki tumor cell lines [49] when compared to primary keratinocytes (Fig. 3a). These results indicate that MYPOP is eliminated in HPV-transformed tumor cells on a post-transcriptional level. This observation was further verified by immunohistochemistry of human cervical carcinoma in situ, where MYPOP expression is present in the keratinocytes of non-lesional cervical tissue (Suppl. Figure 4a), whereas MYPOP expression is almost absent in the carcinoma and only detectable in the non-transformed peripheral keratinocytes (Fig. 3b).

### The HPV16 onco-protein E7 induces downregulation of MYPOP

The high-risk HPV E6 or E7 oncoproteins are potential candidates to induce MYPOP downregulation. These proteins are highly expressed in HPV-induced cancers and possess the ability to target tumor suppressors for degradation [5, 30, 50–52]. Importantly, our quantitative western blot analyses demonstrated that overexpression of HPV16 E6 protein had no effect on MYPOP protein amounts (Fig. 3c), whereas HPV16 E7 significantly reduced MYPOP expression levels (Fig. 3d). Together these data provide strong indication that the absence of MYPOP detected in HPV-transformed tumor cell lines and in cervical cancer tissues results from the expression of the papillomaviral oncogene E7.

### MYPOP suppresses colony formation of tumor cells

**Fig. 4:**
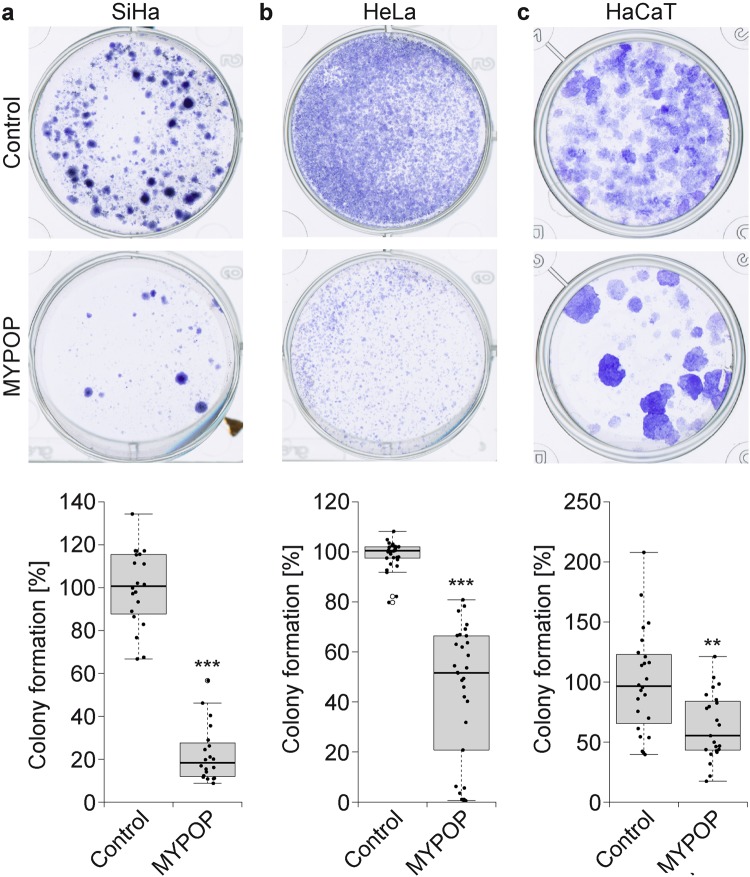
MYPOP inhibits colony formation of HPV-transformed and non-virally transformed cells. **a–c** Cells were transfected with either MYPOP expression plasmid or a control plasmid and selected for 6–12 days with G418. Colonies of control or MYPOP-transfected cells were fixed with methanol and stained using crystal violet (upper panel **a–c**). Plates were quantified using ImageJ plugin “ColonyArea” (lower panel **a–c**) and values are given as boxplots. **a** Shown are representative image of SiHa cells and the values obtained from five independent experiments. Control-transfected cells were set to 100%. Data (*n* = 20) were analyzed using Wilcoxon rank sum test *p* = 1.451 × 10^−**11**^, *W* = 400. **b** Shown are representative image of HeLa cells and the values obtained from nine independent experiments. Control-transfected cells were set to 100%. Data (*n* = 30) were analyzed using Wilcoxon rank sum test *p* = 2.2 × 10^−**16**^, *W* = 899. **c** Shown are representative image of HaCaT cells and the values obtained from six independent experiments. Control-transfected cells were set to 100%. Data (*n* = 22) were analyzed using Welch two-tailed *t*-test *p* = 0.00105, *t* = 3.5361, dF = 39.71; **p ≤ 0.01, ***p ≤ 0.001

The maintenance of early gene expression is a precondition for proliferation and survival of HPV-induced cancer cells [53–55] and seems to be linked to the decreased MYPOP levels. On the other hand, re-expression of MYPOP might result in reduced cell growth. We therefore overexpressed MYPOP in HPV-transformed SiHa and HeLa cells and tested arrest of proliferation in microscopy analyses or standard quantitative colony formation assays, as described previously [56, 57]. Indeed, MYPOP-GFP-expressing cells displayed altered cell morphology and no tendency to form colonies (Suppl. Figure 4b). Furthermore, expression of MYPOP significantly reduced the number of cells measured by analyzing the area of formed colonies (Fig. 4a, b). MYPOP and GFP-MYPOP expression displayed comparable effects in the colony formation assays. Taken together, our data provide strong indication that MYPOP act as a tumor suppressor in HPV-induced cancer.

MYPOP’s repressive activity might be explained by competition of MYPOP with activating TFs. For c-Myb, it has been shown earlier that it binds to one MRE in the HPV16 LCR and activates early gene expression [11]. Therefore, we conclude that c-Myb and MYPOP might be opponents in HPV-associated carcinogenesis as (i) both proteins bind to the same DNA motifs, (ii) cause contradicting effects on the HPV16 LCR, and (iii) are inversely regulated (c-Myb protein level is elevated and MYPOP is reduced) in HPV-transformed cells. As c-Myb has been described as proto-oncogene [58], MYPOP might function as a more general tumor suppressor. Indeed, the expression of MYPOP in non-virally transformed HaCaT cells led to a significant reduction of formed colonies (Fig. 4c), supporting the anti-proliferative tumor suppressor function of the Myb-related TF MYPOP.

Overall, our study provides first indications for the so far unknown roles of MYPOP by demonstrating that this Myb-related protein senses incoming viruses and represses viral gene transcription. Thereby, MYPOP acts as a restriction factor and limits cells’ permissiveness to infection with oncogenic HPV viruses. Mechanistically, we propose a model in which MYPOP senses incoming oncogenic HPV by interaction with the accessible cytoplasmic part of the L2 protein until release of the viral DNA within the nucleus. Subsequently, MYPOP binds to the LCR via its DBD. This results in silencing of HPV early/oncogene expression and subsequently, suppression of cancer. During cell transformation, the HPV16 onco-protein E7 mediates degradation of MYPOP by a mechanism that is yet to be determined, which results in increased expression of the early viral genes, cell proliferation, and finally oncogenesis. A detailed investigation and elucidation of this transcriptional repressor will be crucial for better understanding of infections by oncogenic papillomaviruses and tumor suppression in general.

## Electronic supplementary material

Supplementary figures

Supplementary methods
