# Supplementary material for: The Myb-related protein MYPOP is a novel intrinsic host restriction factor of oncogenic human papillomaviruses
Source: Oncogene. 2018 Jul 17;37(48):6275–84. doi: 10.1038/s41388-018-0398-6 (PMC6265261; doi:10.1038/s41388-018-0398-6)
Supplement: Supplementary file 1 — Supplementary figures [file 41388_2018_398_MOESM1_ESM.docx]

**SUPPLEMENTARY INFORMATION**

**The Myb-related protein MYPOP is a novel intrinsic host restriction factor of oncogenic human papillomaviruses**

Elena Wüstenhagen^1^, Fatima Boukhallouk^1^, Inka Negwer^2^, Krishnaraj Rajalingam^3^, Frank Stubenrauch^4^, and Luise Florin^1,^*

^1^Department of Medical Microbiology and Hygiene, University Medical Center of the Johannes Gutenberg University Mainz, Obere Zahlbacher Strasse 67, 55131 Mainz, Germany; ^2^Max Planck Institute for Polymer Research, Ackermannweg 10, 55128 Mainz, Germany; ^3^Molecular Signaling Unit, University Medical Center of the Johannes Gutenberg University Mainz; ^4^Division of Experimental Virology, Institute for Medical Virology and Epidemiology of Viral Diseases, University Hospital Tübingen, Germany

*correspondence should be addressed to lflorin@uni-mainz.de

**Suppl. Fig. 1** MYPOP antibody evaluation for immunofluorescence and immunodetection.

**a, b** Comparison of both MYPOP antibodies for their reactivity after methanol or 4% PFA-0.2% Triton X-100 fixation in immunofluorescence. HaCaT cells were transiently transfected for 24 h using either untagged (**a**) or GFP- tagged MYPOP (**b**). The cells were fixed either by methanol or 4% PFA-0.2% Triton X-100 and stained using either polyclonal rabbit ProteoGenix antibody or polyclonal rabbit Abcam antibody as indicated (magenta). Nuclei are shown with dotted lines or in blue. Scale bar = 10 µm. The MYPOP-ProteoGenix antibody displayed the higher sensitivity in IF studies and the MYPOP-Abcam antibody in WB analyses. Therefore, we used the MYPOP-ProteoGenix antibody in IF and the MYPOP-Abcam antibody in WB experiments.

**c** Comparison of both MYPOP antibodies and their reactivity in immunoblot. HeLa, HaCaT and primary keratinocytes cell lysates together with purified MYPOP-Myc-his_6_ were separated by SDS-PAGE, processed by Western blot and analyzed by immunodetection. Detection of MYPOP was performed using either ProteoGenix or Abcam antibody. The arrow indicates the main band. Due to clarity and conciseness the Western blot images are cropped.

**Suppl. Fig. 2** Analysis of the MYPOP DNA binding domain and the Myb recognition elements in the HPV LCR.

**a-c** MYPOP DNA binding domain is sufficient for repression activity.

**a** Schematic representation of human MYPOP-WT and the two deletion mutants, MYPOP-N (aa 1-126) and MYPOP-C (aa 127 to 400) and its predicted protein domains. DBD = DNA binding domain; NLS = nuclear localization signal; NES = nuclear export signal

**b** HaCaT cells were transiently co-transfected with pGL4.20 HPV16 LCR luciferase reporter plasmid together with control FLAG vector or FLAG-MYPOP for 24 hours. Cells were then lysed to monitor MYPOP expression by Western blot using monoclonal mouse FLAG antibody. The lower panel shows an unspecific band as loading control.

**c** HaCaT cells were transiently transfected with pGL4.20 HPV16 LCR and empty FLAG vector, MYPOP-WT, MYPOP-N or MYPOP-C for 24 hours. Influence of MYPOP and its mutants on LCR was measured by luciferase activity and normalized to lactate dehydrogenase (LDH) activity. The values obtained from four independent experiments are given as boxplots and pGL4.20 16 LCR with empty FLAG vector was set to 100%. Data (n=15) were analyzed using two-tailed unpaired t-test: p=4.475x10^-9^, t=8.3421, dF=28 (MYPOP-WT), p=5.016x10^-7^, t=6.4848, dF=28 (MYPOP-N), p=0.08089, t=1.8109, dF=28 (MYPOP-C); ns, not significant; ***, p≤0.001

**d-e** Myb recognition elements in the HPV LCR.

**d** Schematic representation of the full length HPV16 LCR (16LCR) and deletion constructs thereof. Black lines represent putative Myb recognition elements (MRE).

**e** All LCR fragments containing at least one binding site were repressed by MYPOP. HaCaT cells were transiently co-transfected with the empty pGL4.20 or pGL4.20 HPV16 LCR or LCR mutants, which were shortened from the 5’-end (as indicated) together with FLAG-MYPOP for 24 hours. Influence of MYPOP on empty pGL4.20 luciferase reporter plasmid or pGL4.20 luciferase reporter plasmid containing full-length HPV16 LCR or LCR mutants was measured by luciferase activity and normalized to lactate dehydrogenase (LDH) activity. The values obtained from six independent experiments are given as boxplots and the corresponding pGL4 LCR plasmid with empty FLAG vector was set to 100%. Data were analyzed using Welch two-tailed t-test: p=1.561x10^-8^, t=8.3763, dF=23.637 (16LCR 7157-94, n=25), p=2.472x10^-8^, t=8.0245, dF=24.636 (16LCR 7540-94, n=29), p=1.089x10^-8^, t=8.4993, dF=23.93 (16LCR 7571-94, n=29), p=8.443x10^-7^, t=6.6591, dF=23.066 (16LCR 7777-94, n=25) or Wilcoxon rank sum test p=0.3107, W=216 (16LCR 15-94, n=19). ns, not significant; ***, p≤0.001.

**f** Schematic representation of LCR of HPV11, HPV16 and HPV18. Black lines represent putative MREs.

**Suppl. Fig. 3** MYPOP siRNA treatment of HaCaT cells for 48 hours.

**a** Cells were transfected with control siRNA or MYPOP-specific siRNAs for 48 hours. Then, total mRNA was isolated, reverse transcribed to cDNA, and analyzed by quantitative real-time PCR. The values obtained from three independent experiments are given as boxplots and control siRNA-treated cells were set to 100%. Data were analyzed using Welch two-tailed t-test: p=1.166x10^-6^, t=13.912, dF=7.5639 (#9, n=5), p=2.329x10^-6^, t=18.4, dF=5.7911 (#10, n=6), p=0.05926, t=2.3299, dF=5.9149 (#11, n=6), or two-tailed unpaired t-test: p=2.281x10^-6^, t=9.6122, dF=10 (#12, n=6), p=4.203x10^-8^, t=14.715, dF=10 (pool, n=6). ns, not significant; ***, p≤0.001

**b** Same as panel **a**, but cells were lysed to monitor knockdown efficiency of MYPOP by Western blot using polyclonal rabbit MYPOP antibody.

**c** Same as panel **a**, but cells were infected with HPV16 LCR PsV. Luciferase activity as measure for infection was assessed 24 hours later and normalized to LDH measurements. The values obtained from two independent experiments are given as boxplots and control siRNA-treated cells were set to 100%. Data (n=8) were analyzed using two-tailed unpaired t-test: p=0.3594, t=-0.94756, dF=14 (#9), p=0.9783, t=0.02775, dF=14 (#10), p=0.1713, t=-1.4421, dF=14 (#11), p=0.0292, t=-2.4289, dF=14 (#12), p=0.09665, t=-1.7808, df=14 (pool). *, p≤0.05; ns, not significant

**Suppl. Fig. 4**

**a** MYPOP is expressed in normal cervical keratinocytes. Human cervical tissue sections were stained for MYPOP (green). Nuclei were counterstained with Hoechst (blue). Scale bar = 10 µm

**b** MYPOP overexpression alters cell morphology of HPV transformed cell lines. HeLa and SiHa cells were transfected with either MYPOP expression plasmid or a control plasmid for 24 h and transfected cells were selected for 6-12 day with G418. GFP or GFP-MYPOP expressing cells were analyzed by fluorescence microscopy.
