# Supplementary material for: The Myb-related protein MYPOP is a novel intrinsic host restriction factor of oncogenic human papillomaviruses
Source: Oncogene. 2018 Jul 17;37(48):6275–84. doi: 10.1038/s41388-018-0398-6 (PMC6265261; doi:10.1038/s41388-018-0398-6)
Supplement: Supplementary file 2 — Supplementary methods [file 41388_2018_398_MOESM2_ESM.docx]

**SUPPLEMENTARY METHODS**

**The Myb-related protein MYPOP is a novel intrinsic host restriction factor of oncogenic human papillomaviruses**

Elena Wüstenhagen^1^, Fatima Boukhallouk^1^, Inka Negwer^2^, Krishnaraj Rajalingam^3^, Frank Stubenrauch^4^, and Luise Florin^1,^*

^1^Department of Medical Microbiology and Hygiene, University Medical Center of the Johannes Gutenberg University Mainz, Obere Zahlbacher Strasse 67, 55131 Mainz, Germany; ^2^Max Planck Institute for Polymer Research, Ackermannweg 10, 55128 Mainz, Germany; ^3^Molecular Signaling Unit, University Medical Center of the Johannes Gutenberg University Mainz; ^4^Division of Experimental Virology, Institute for Medical Virology and Epidemiology of Viral Diseases, University Hospital Tübingen, Germany

*correspondence should be addressed to lflorin@uni-mainz.de

**Cell lines and transfection.** HaCaT cells (human non-virally transformed keratinocytes) were obtained from Cell Lines Services (CLS), Eppelheim, Germany. The human cervical carcinoma cell line HeLa was purchased from the German Resource Center of Biological Material (DSMZ, Braunschweig, Germany). The human cervical carcinoma cell lines SiHa and CaSki were kindly provided by W. Zwerschke, Institute for Biomedical Aging Research, Innsbruck, Austria. All cell lines were cultivated as described in^1,2^. Normal Human Epidermal Keratinocytes (NHEK) were purchased from PromoCell, Heidelberg, Germany and cultivated according to the manufacturer’s instructions. SCC-13 cells (derived from a squamous cell carcinoma of the cheek) were kindly provided by L.A. Laimins, Northwestern University Medical School, Chicago, IL, USA and were maintained in E-medium supplemented with 5% fetal bovine serum in the presence of mitomycin C-treated NIH3T3 J2 fibroblasts. Cells were either transfected using polyethyleneimine (Sigma Aldrich St. Louis, MO, USA), Fugene HD (Promega, Frichburg, WI, USA), Lipofectamine 2000 or RNAiMAX (both Thermo Fisher Scientific, Waltham, MA, USA) according to manufacturer’s protocol.

**Plasmids.** The pCMV-AC-GFP-MYPOP plasmid was purchased from OriGene, Rockville, MD, USA. To construct full length FLAG-tagged, and EGFP-tagged and untagged MYPOP expression vector, MYPOP was amplified using the pCMV-AC-GFP-MYPOP expression plasmid as template with primers listed in Suppl. table 1 for plasmid construction. The mutants, MYPOP-N (AA 1 to 126) and MYPOP-C (AA 127 to 400) were constructed and cloned by using specific primers listed in Suppl. table 1 for plasmid construction. All three MYPOP fragments (full length, MYPOP-N and MYPOP-C) fragment were cloned into p3xFLAG-CMV-10 (Sigma Aldrich, St. Louis, MO, USA). EGFP-tagged and untagged MYPOP was cloned into pEGFP-C3 (Clontech, Mountain View, CA, USA) or pcDNA3.1(+) (Invitrogen, Carlsbad, CA, USA). All constructs were confirmed by sequencing. Codon-optimized HPV16 L1 and L2 expression plasmids were prepared based on the wild-type origin vector described before^3^. HA-tagged HPV16 E6 expression vector was kindly provided by Claudia Simon, Tübingen, Germany. The expression plasmid encoding FLAG/HA-tagged HPV16 E7 (Addgene plasmid 13734) was kindly provided by Karl Munger, Boston, MA, USA. The promoter reporter plasmids pGL4.20 HPV11 LCR (Addgene plasmid 22858) and pGL4.20 HPV18 LCR (Addgene plasmid 22859) were kindly provided by Peter M. Howley, Boston, MA, USA. pGL4.20 HPV16 LCR has been described earlier^4^. The mutants 16LCR 7540-94, 16LCR 7571-94, 16LCR 7777-94 and 16LCR 15-94 were constructed by PCR, restricted with the respective enzymes and cloned into pGL4.20 puro. Target sequences are available on request.

**Antibodies.** For detection of FLAG-tagged MYPOP after Western blot or immunofluorescence staining, either monoclonal mouse antibody M2 (Sigma Aldrich, St. Louis, MO, USA) or polyclonal rabbit antibody (Rockland, Limerick, PA, USA) were used. HPV16 L2-specific mouse antibody 33L2-1 have been previously described^5^. HA-specific mouse antibody was purchased from Covance (Princeton, NJ, USA). MYPOP-specific polyclonal rabbit antibody (ab94406) was purchased from Abcam, Cambridge, UK. Additionally, a polyclonal serum against the peptide sequence KRTGQEVQKRWND-cys was generated to detect MYPOP (ProteoGenix, Schiltigheim, France). β-actin (A5441)-specific mouse antibody and control mouse IgG antibody were purchased from Sigma Aldrich (St. Louis, MO, USA). HRP-coupled secondary antibodies were purchased from Dianova (Hamburg, Germany), secondary antibodies for immunofluorescence detection (AlexaFluor®) as well as the Click-iT® EdU Imaging Kit were purchased from Invitrogen, Carlsbad, CA, USA.

**Yeast two-hybrid screening.** The yeast two-hybrid screening with L2 as bait using a cDNA library (pJG4-5) derived from human serum-starved WI-38 fibroblasts has been described previously^4,6^.

**Co-immunoprecipitations.** For coimmunoprecipitation, HaCaT cells were seeded in 10 cm dishes and transfected with the appropriate plasmids for 24 h using polyethylenimine. Thereafter, cells were lysed using MACS lysis buffer (MACS Miltenyi Biotec, Bergisch Gladbach, Germany), containing dithiothreitol (DTT) and protease inhibitors Aprotinin and Leupeptin (10 µg/ml each), and incubated for 20 min at 4°C on an overhead rotator. Lysates were treated 3 times with an ultrasonicator (30% duty cycle; output control, 30%; Branson Sonifier 250, Emerson Industrial Automation, St. Louis, MO, USA) for 20 s and incubated for 20 min at 4°C on an overhead rotator. Lysates were precleared with 50 µl protein A/G agarose (Santa Cruz Biotechnology, Dallas, TX, USA). Precleared lysates were incubated with either 1 µl anti-FLAG antibody (Sigma Aldrich, St. Louis, MO, USA) for precipitation of FLAG-MYPOP or 40 µl L2-1 for precipitation of L2 for 1 h at 4°C on a rotating wheel and for 1 h after addition of 50 µl protein A/G agarose. Agarose was washed with wash buffer containing 500 mM NaCl, 50 mM Tris-HCl (pH 8.0), 1% (v/v) NP40 (Sigma Aldrich, St. Louis, MO, USA), 0.5% (w/v) sodium deoxycholate (Sigma Aldrich, St. Louis, MO, USA), 0.1% SDS (Carl Roth, Karlsruhe, Germany) supplemented with protease inhibitors. The precipitates were boiled in SDS sample buffer and processed for Western blot.

**Production of pseudoviruses (PsV).** HPV16 PsV and HPV16 EdU-modified PsV were prepared as previously described^7,8^ using pGL4.20 HPV16 LCR as maker plasmid and termed as HPV16 LCR PsV. Quantification of the pGL4.20 HPV16 LCR-positive pseudovirions was performed by quantitative PCR using SYBR green and the primer listed in Suppl. table 1. The qPCR was performed with a 7500 Real-Time PCR System and Sequence Detection Software v2.3 (Applied Biosystems, Foster City, CA, USA).

**Immunofluorescence microscopy.** HaCaT cells were grown on coverslips. After transfection and/or infection with 500 HPV16 viral genome equivalents (vge), cells were washed and fixed with ice-cold methanol at -20°C for 2-10 min or 30 min according to EdU staining protocol or with 4% paraformaldehyde followed by permeabilization with PBS containing 0.2% Triton X-100. Staining of viral DNA was performed according to the manufacturer’s protocols (Thermo Fisher Scientific, Waltham, MA, USA). Fixed cells were washed with phosphate-buffered saline (PBS) and blocked for 30 min with PBS, 1% (w/v) bovine serum albumin (BSA, AppliChem, Darmstadt, Germany). Afterwards, proteins were stained with indicated antibodies at 37°C for 1 h. After washing and blocking, cells were incubated with Alexa-conjugated specific secondary antibodies for 1 h at 37°C. DNA was counterstained using Hoechst33342 (Invitrogen, Carlsbad, CA, USA). Coverslips were mounted onto slides using Fluoprep mounting medium (bioMérieux, Marcy-I’Étoile, France). Fluorescence imaging was performed on a Zeiss Axiovert 200 M microscope and Z-stack images were deconvoluted using the software supplied by Zeiss (Axiovision 4.7, Carl Zeiss, Jena, Germany).

**Confocal Laser Scanning Microscopy**. Images were recorded with a LSM880 confocal laser scanning microscope (Carl Zeiss, Jena, Germany) using a water immersion objective (C-Apochromat 40x/1.2 W). Alexa Fluor®488-clicked vDNA was excited with the 488 nm line of an Argon laser and the fluorescence was detected in the wavelength range 500 - 553 nm. MYPOP-FLAG (Alexa Fluor®546-labeled secondary antibody) and L2 (Alexa Fluor®647-labeled secondary antibody) were excited with HeNe lasers at 543 nm and 633 nm and the emission channels were set to 562 - 633 nm and 642 - 696 nm, respectively. In each experiment a z-stack with a distance of 0.5 µm between the slices was acquired and a representative slice for which the structures were best in focus was selected.

**Immunostaining of tissue slices.** Paraffin-embedded cervix tissue slices were stained as described previously^9,10^.

**RNAi-mediated knockdown and HPV16 PsV infection assay.** The MYPOP-specific siRNAs, as well as the non-silencing control siRNA were purchased from Qiagen, Hilden, Germany. The following gene-specific siRNAs were used: MYPOP: Hs_LOC339344_9/10/11/12. Cells were transfected with 30 nM siRNA using Lipofectamine RNAiMAX according to the manufacturer’s instructions (Thermo Fisher Scientific, Waltham, MA, USA).

Subsequent experiments were performed 48 h or 96 h after siRNA transfection using HaCaT cells. For 96 h siRNA transfection, cells were re-transfected after 48 h with siRNA. After siRNA-mediated knockdown, cells were exposed to ≈200 vge and infection was measured as published before by determining relative luciferase activity^11^ (for relative luciferase measurement see also promoter-reporter assay). The siRNA knockdown efficiency and the endogenous MYPOP level in different cell lines were quantified by Western blot and quantitative Polymerase Chain Reaction (qPCR). qPCR experiments were performed as described previously^1^. The qPCR was performed with a 7300 or 7500 Real-Time PCR System and Sequence Detection Software 4.0 (Applied Biosystems, Foster City, CA, USA) and analyzed by using the comparative cycle threshold (c_T_) method.

For lentivirally transduced MYPOP-specific shRNA, Mission® TRC2 pLKO.5-puro vector carrying either no shRNA or MYPOP-specific shRNA was transfected into HEK293T cells, along with packaging vectors, using Lipofectamine 2000 transfection reagent (Thermo Fisher Scientific, Waltham, MA, USA). Medium was changed 24 hours post transfection, and cells were allowed to grow for additional 24 h. Supernatants were harvested, filtrated through a 0.45 µm filter and used immediately for infection. Each filtrated supernatant was supplemented with cell culture medium containing 20% FCS and 8 µg/ml Polybrene (Sigma Aldrich, St. Louis, MO, USA), which was immediately added to the recipient HaCaT cells. Medium was chanced 24 hours post transduction and incubated for additional 24 hours. Cells were then selected for stably silenced cell lines using puromycin (5 µg/ml). Knockdown was confirmed by Western blot analysis and infection assay was performed.

**Chromatin immunoprecipitation (ChIP).** Chromatin immunoprecipitations were conducted as previously described^4^. Chromatin preparation and immunoprecipitation were performed with the SimpleChIP Enzymatic Chromatin IP Kit (Cell Signaling, Cambridge, UK) according to the manufacturer’s instructions. Chromatin was sheared using a Bioruptor ultrasonic bath (Diagenode, Seraing (Ougrée), Belgium). The precipitated DNA fragments were used as templates for PCR amplification to detect co-precipitation of the HPV18 LCR. A fragment spanning from nucleotide (nt) 7735 to nt 124 of the HPV18 genome was amplified using a primer set listed in Suppl. table 1. Precipitated DNA fragments were analyzed by qPCR using a 7300 real-time PCR system and Sequence Detection Software 4.0 (Applied Biosystems, Foster City, CA) or 7500 Real-Time PCR System and Sequence Detection Software 4.0 (Applied Biosystems, Foster City, CA, USA) and analyzed using the percent input method.

**Native affinity purification of MYPOP-his_6_.** For purification of human MYPOP-his_6_, HEK293T cells were seeded in 15 cm dishes and transfected with the appropriate MYPOP-plasmid for 48 h using polyethylenimine. MYPOP-his_6_ was extracted using xTractor Buffer (Clontech, Mountain View, CA, USA) followed by an additional sonification step on ice (5 times for 20 sec, 30% duty cycle and 30% output control). Protein purification was performed under native conditions using His60 Ni Superflow Resin according to the manufacturer’s protocol (Clontech, Mountain View, CA, USA). Purified protein was boiled in SDS sample buffer and processed for Western blot.

**Promoter reporter gene assay.** HaCaT cells were seeded into 24 well plates 24 hours prior transfection. Cells were co-transfected using polyethyleneimine with a total of 1 µg DNA, composed of equal amounts of luciferase reporter vector and protein expression vector. In dose-dependent promoter-reporter gene assays, decreasing amounts of expression vector was replaced with control vector. After 24 hours, the cells were lysed using Luciferase Cell Culture Lysis Reagent (Promega, Frichburg, WI, USA) and luciferase was measured and normalized by lactate dehydrogenase (LDH) measurements (CytoTox-ONE homogeneous membrane integrity assay, Promega, Frichburg, WI, USA). Luciferase and LDH activities were measured with a Tristar LB 941 luminometer (Berthold Technologies, Bad Wildbad, Germany).

**Whole genome assay**. SCC13 cells (6x10^5^) were seeded into 35 mm dishes the day before transfection. The next day, 1.3 µg re-circularized HPV16 genomes (isolate 114B; Genbank: KU053852.1) were co-transfected with 0.5 µg of MYPOP-FLAG or empty FLAG vector using Fugene HD (Promega, Frichburg, WI, USA). RNA was isolated 48h later using the RNeasy Mini Kit (Qiagen, Hilden, Germany). RNA was reverse transcribed using the QuantiTect reverse transcription kit (Qiagen, Hilden, Germany) and cDNA aliquots were analyzed by qPCR using 0.3 µM gene-specific primers and 1xLightCycler 480 SYBR green I Master (Roche Diagnostics Deutschland GmbH, Mannheim, Germany). Primers oCCB-396 and oCCB-350 were used to detect the spliced HPV16 E6*I transcript and primers AM1132 and AM1133 to detect spliced HPV16 E1^E4 transcripts^12^. HPV16 transcript levels were normalized to PGK1 transcript levels as previously described^13^.

**Colony Formation Assay.** Cells were seeded into 6 or 12 well plates and transfected with the appropriate plasmids for 24 h using polyethylenimine. Fluorescence microscopy analyses showed reproducible and comparable transfection efficiencies. Transfected cells were selected for 6 to 12 days in 1.2 mg/ml G418 (Carl Roth, Karlsruhe, Germany). Non-transfected cells were used as an internal control for successful selection. Colonies were fixed with methanol and stained with Crystal violet as described previously^14-17^. Plates were scanned and analyzed using ImageJ plugin “ColonyArea”^18^.

**Statistics.** Data analysis was performed using Statistical Software R (2017, version 3.3.1) from R Core Team (R: A language and environment for statistical computing. R Foundation for Statistical Computing, Vienna, Austria). All values were tested for normality using Shapiro-Wilk test. If the values followed normal distribution, they were further tested for homogeneity of variances using Bartlett, Fligner-Killeen and Levene‘s test. Differences between the groups for homoscedastic samples were analyzed using unpaired two-tailed t-test and for heteroscedastic with Welch two-tailed t-test. In the case that values were not normally distributed, Wilcoxon rank sum test was applied. Differences between the groups were considered statistically significant when p≤0.05 with the statistical significance marked in the graph (p≤0.05 *, p≤0.01 **, p≤0.001 ***, ns=not significant).

**Supplementary table 1**

**Supplementary References:**

1 Wüstenhagen E, Hampe L, Boukhallouk F, Schneider MA, Spoden GA, Negwer I *et al.* The Cytoskeletal Adaptor Obscurin-Like 1 Interacts with the Human Papillomavirus 16 (HPV16) Capsid Protein L2 and Is Required for HPV16 Endocytosis. 2016; **90**: 10629–10641.

2 Gräßel L, Fast LA, Scheffer KD, Boukhallouk F, Spoden GA, Tenzer S *et al.* The CD63-Syntenin-1 Complex Controls Post-Endocytic Trafficking of Oncogenic Human Papillomaviruses. *Sci Rep* 2016; **6**: 32337.

3 Leder C, Kleinschmidt JA, Wiethe C, MÜLLER M. Enhancement of capsid gene expression: preparing the human papillomavirus type 16 major structural gene L1 for DNA vaccination purposes. 2001; **75**: 9201–9209.

4 Schneider MA, Scheffer KD, Bund T, Boukhallouk F, Lambert C, Cotarelo C *et al.* The Transcription Factors TBX2 and TBX3 interact with HPV16 L2 and repress the Long Control Region of Human Papillomaviruses. 2013. doi:10.1128/JVI.01803-12.

5 Volpers C, Sapp M, Snijders PJ, Walboomers JM, Streeck RE. Conformational and linear epitopes on virus-like particles of human papillomavirus type 33 identified by monoclonal antibodies to the minor capsid protein L2. *J Gen Virol* 1995; **76 ( Pt 11)**: 2661–2667.

6 Schneider MA, Spoden GA, Florin L, Lambert C. Identification of the dynein light chains required for human papillomavirus infection. *Cell Microbiol* 2011; **13**: 32–46.

7 Buck C, Pastrana D, Lowy D, Schiller J. Efficient intracellular assembly of papillomaviral vectors. 2004; **78**: 751.

8 Bund T, Spoden GA, Koynov K, Hellmann N, Boukhallouk F, Arnold P *et al.* A L2 SUMO Interacting Motif is Important for PML-Localization and Infection of Human Papillomavirus Type 16. *Cell Microbiol* 2014. doi:10.1111/cmi.12271.

9 Florin L, Sapp C, Streeck R, Sapp M. Assembly and translocation of papillomavirus capsid proteins. 2002; **76**: 10009.

10 Florin L, Becker KA, Sapp C, Lambert C, Sirma H, Müller M *et al.* Nuclear translocation of papillomavirus minor capsid protein L2 requires Hsc70. 2004; **78**: 5546–5553.

11 Spoden GA, Besold K, Krauter S, Plachter B, Hanik N, Kilbinger AFM *et al.* Polyethylenimine Is a Strong Inhibitor of Human Papillomavirus and Cytomegalovirus Infection. *Antimicrobial Agents and Chemotherapy* 2011; **56**: 75–82.

12 Soeda E, Ferran MC, Baker CC, McBride AA. Repression of HPV16 early region transcription by the E2 protein. *Virology* 2006; **351**: 29–41.

13 Straub E, Dreer M, Fertey J, Iftner T, Stubenrauch F. The viral E8^E2C repressor limits productive replication of human papillomavirus 16. 2014; **88**: 937–947.

14 Franken NAP, Rodermond HM, Stap J, Haveman J, van Bree C. Clonogenic assay of cells in vitro. *Nat Protoc* 2006; **1**: 2315–2319.

15 Feoktistova M, Geserick P, Leverkus M. Crystal Violet Assay for Determining Viability of Cultured Cells. *Cold Spring Harb Protoc* 2016; **2016**: pdb.prot087379.

16 van Doorslaer K, Chen D, Chapman S, Khan J, McBride AA. Persistence of an Oncogenic Papillomavirus Genome Requires cis Elements from the Viral Transcriptional Enhancer. *MBio* 2017; **8**: e01758–17.

17 Leitz J, Reuschenbach M, Lohrey C, Honegger A, Accardi R, Tommasino M *et al.* Oncogenic Human Papillomaviruses Activate the Tumor-Associated Lens Epithelial-Derived Growth Factor (LEDGF) Gene. *PLoS Pathog* 2014; **10**: e1003957.

18 Guzmán C, Bagga M, Kaur A, Westermarck J, Abankwa D. ColonyArea: an ImageJ plugin to automatically quantify colony formation in clonogenic assays. *PLoS ONE* 2014; **9**: e92444.
